# Ultrastrong Hybrid Fibers with Tunable Macromolecular Interfaces of Graphene Oxide and Carbon Nanotube for Multifunctional Applications

**DOI:** 10.1002/advs.202203008

**Published:** 2022-08-21

**Authors:** Seo Gyun Kim, So Jeong Heo, Jeong‐Gil Kim, Sang One Kim, Dongju Lee, Minkook Kim, Nam Dong Kim, Dae‐Yoon Kim, Jun Yeon Hwang, Han Gi Chae, Bon‐Cheol Ku

**Affiliations:** ^1^ Institute of Advanced Composite Materials Korea Institute of Science and Technology (KIST) Wanju 55324 Republic of Korea; ^2^ Department of Materials Science and Engineering Ulsan National Institute of Science and Technology (UNIST) Ulsan 44919 Republic of Korea; ^3^ Department of Chemical and Biomolecular Engineering Korea Advanced Institute of Science and Technology (KAIST) Daejeon 34141 Republic of Korea; ^4^ Department of Carbon Materials and Fiber Engineering Jeonbuk National University Jeonju 54896 Republic of Korea; ^5^ Department of Applied Bioengineering Graduate School of Convergence Science and Technology Seoul National University Suwon 16229 Republic of Korea; ^6^ Department of Nano Convergence Jeonbuk National University Jeonju 54896 Republic of Korea

**Keywords:** carbon nanotube fibers, graphene oxide, hybrid fibers, multidimensional nanostructure, wet spinning

## Abstract

Individual carbon nanotubes (CNT) and graphene have unique mechanical and electrical properties; however, the properties of their macroscopic assemblies have not met expectations because of limited physical dimensions, the limited degree of dispersion of the components, and various structural defects. Here, a state‐of‐the‐art assembly for a novel type of hybrid fiber possessing the properties required for a wide variety of multifunctional applications is presented. A simple and effective multidimensional nanostructure of CNT and graphene oxide (GO) assembled by solution processing improves the interfacial utilization of the components. Flexible GOs are effectively intercalated between nanotubes along the shape of CNTs, which reduces voids, enhances orientation, and maximizes the contact between elements. The microstructure is finely controlled by the elements content ratio and dimensions, and an optimal balance improves the mechanical properties. The hybrid fibers simultaneously exhibit exceptional strength (6.05 GPa), modulus (422 GPa), toughness (76.8 J g^–1^), electrical conductivity (8.43 MS m^–1^), and knot strength efficiency (92%). Furthermore, surface and electrochemical properties are significantly improved by tuning the GO content, further expanding the scope of applications. These hybrid fibers are expected to offer a strategy for overcoming the limitations of existing fibers in meeting the requirements for applications in the fiber industry.

## Graphical abstract

Wet spinning with CSA facilitates the dispersion of CNT and GO and the orientation and packing of fibers. The microstructures are controlled by the dimensions and content ratio of the components. Desirable structuring dramatically improves the mechanical properties by improving the alignment, reducing voids, and maximizing interfacial contact. Extraordinary flexibility, surface and electrochemical properties make them attractive for multifunctional applications.

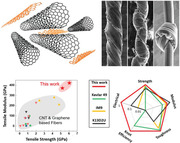

## 1 Introduction

High‐performance synthetic fibers are commonly composed of long macromolecules that are highly aligned along the fiber axis.^[^ ^1^ ^]^ 1D carbon nanotubes (CNTs) and 2D graphene with a high aspect ratio have been considered the ultimate building blocks for high‐performance assemblies because of their excellent mechanical, electrical, and thermal properties, including flexibility.^[^ ^2^, ^3^, ^4^, ^5^, ^6^ ^]^ Their impressive combination of properties has attracted considerable research interest for various applications.^[^ ^7^, ^8^, ^9^, ^10^ ^]^ However, the bulk properties of CNT‐ or graphene‐based assemblies are an obstacle to exploiting the desirable properties of individual CNT and graphene particles, because of the imperfect alignment of particles, the presence of pores, and the limited connectivity of elements in the assemblies.^[^ ^6^, ^7^, ^11^, ^12^ ^]^ To address these problems, many studies have been conducted to improve fiber properties, using post‐treatment processes,^[^ ^13^, ^14^, ^15^, ^16^ ^]^ structural approaches,^[^ ^6^, ^14^, ^15^, ^16^, ^17^ ^]^ and processing methods^[^ ^11^, ^12^, ^18^ ^]^ for individual elements or assemblies.

In CNT and graphene assemblies, the key problems are to maximize the interactions between the constituents by dispersing the elements and aligning them along the fiber axis.^[^ ^2^, ^19^, ^20^ ^]^ If CNTs or graphene form bundles or graphitic structures inside the assembly, these aggregates can slip on each other under stress, failing to utilize the interfaces of the intact individual elements. Conversely, fully dispersed nanotube and graphene can fully utilize their interfaces and maximize the interaction among neighboring elements. Therefore, it is necessary to prevent the bundling of CNTs and restacking of graphene. Chlorosulfonic acid (CSA) protonates CNT and graphene, making them exist individually.^[^ ^20^, ^21^ ^]^ Such solutions spontaneously form liquid crystals (LCs) above a certain concentration. LC solutions play a significant role in the alignment and dense packing of fiber elements during the spinning process.^[^ ^3^, ^11^, ^12^ ^]^ In practice, wet‐spun CNT fibers formed using a CSA have excellent macroscopic properties.^[^ ^11^, ^12^, ^15^, ^22^ ^]^ However, the properties of the CNT fibers are highly dependent on the length (aspect ratio) of synthesized CNTs.^[^ ^22^, ^23^, ^24^ ^]^ Therefore, a combination of multidimensional structures can be considered to improve the properties of structural assemblies in restricted synthetic CNTs. Flattened collapsed CNTs of 2D structures can achieve greater interactions than circular CNTs, because of the additional graphene–graphene contact across the cores of nanotubes.^[^ ^17^, ^19^ ^]^ Recently, we reported on high‐performance CNT fibers with multidimensional structures assembled by macromolecular coalescence of nanotubes.^[^ ^15^ ^]^

Hybrid and composite assemblies formed from two or more distinct materials have advanced properties that are not found in the individual elements. In addition, desirable structuring by solution processing enables the simple fabrication of strong assemblies. Many studies have been conducted from various perspectives on CNT‐^[^ ^25^, ^26^, ^27^, ^28^, ^29^, ^30^, ^31^ ^]^ and graphene‐based^[^ ^32^, ^33^, ^34^, ^35^, ^36^, ^37^ ^]^ hybrids and/or composite assemblies, and these studies have identified the superior properties and unique advantages of such hybrids. However, the reported mechanical properties tend not to surpass those of high‐performance carbon fibers (CFs). The dispersion and processing problems of CNT and graphene cause imperfect alignment and multiple pores, making it difficult to fabricate a high‐performance assembly.

We prepared LC solutions by dispersing CNTs and graphene oxides (GOs) in CSA, and manufacturing highly ordered and compact GO/CNT hybrid (G‐CNT) fibers by optimizing the spinning process. Structurally, the introduction of 2D GO between well‐aligned CNT fibers makes it possible to achieve a synergistic effect on fiber properties by multidimensional structures, similar to those of collapsed CNTs. GOs with a large number of hydrophilic functional groups can add various functionalities to their assemblies and expand their range of applications, such as their use in energy storage devices. G‐CNT fibers with exceptional mechanical and electrical properties and high flexibility were developed in this study. The microstructure of G‐CNT fibers is controlled by the content ratio and dimensions of individual elements, linking specifically investigated structures and properties. Furthermore, potential applications based on highly improved surface and electrochemical properties of G‐CNT fibers are discussed.

## 2 Results and Discussion

### 2.1 Optimal Wet Spinning

In this study, we manufactured the hybrid fibers of CNT and GO. Here, GO was used in consideration of the excellent dispersion state in the LC solution and improvement of the surface properties of the hybrid fibers. Therefore, thermal or chemical reduction of GO was not performed in this work.

**Figure 1:**
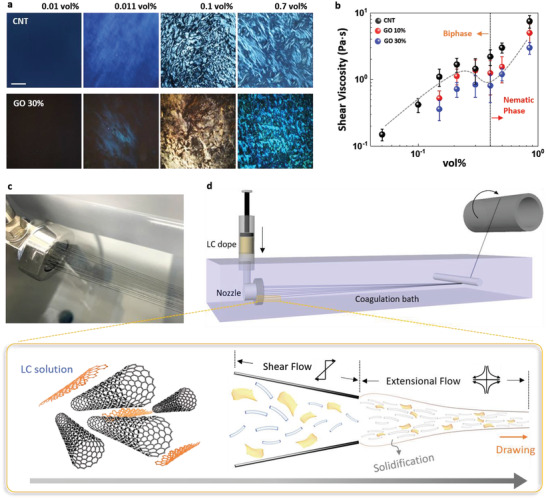
Liquid crystal (LC) behaviors of CNT and CNT/GO solutions, and solution spinning process. a) Polarized optical images of CNT and CNT/GO solutions. The scale bar is 200 µm. b) Shear viscosity of CNT and CNT/GO solutions as a function of solid content at a shear rate of 52 s^–1^. c) Optical image of wet spinning using a 40‐hole nozzle. d) Schematic illustration of wet spinning process for G‐CNT fibers.

Before the fibers were manufactured, the properties of CNT and GO and their LC behaviors were analyzed. The results are shown in **Figure** **1** and Figures S1–S4 (details are provided in the Supporting Information). The LC behavior and spinning process play key roles in determining the structure of assemblies. Carbon‐based anisotropic particles, CNT and graphene, spontaneously form a LC phase in CSA above a certain concentration.^[^ ^20^, ^21^ ^]^ Understanding lyotropic LC can be useful the alignment and macroscopic properties of CNT‐ or graphene‐based assemblies.^[^ ^3^, ^12^, ^38^ ^]^ The shear viscosity of LC solutions of CNT and graphene decreases in the range 0.2–0.4 vol% and then increases (Figure 1b). This behavior change is a phase transition from the biphase to the nematic phase.^[^ ^12^, ^39^ ^]^ This viscosity behavior is also in good agreement with the polarized image of LC solutions (Figure 1a), and similar behaviors have been reported for CNT/sulfuric acid and aqueous GO solutions.^[^ ^40^, ^41^ ^]^ As Figure S4b shows, under the same conditions, the fibers spun in the nematic phase exhibited better properties than those spun in the biphase. A biphase LC solution may exhibit a poorer orientation than a nematic LC solution, which may affect the microstructures and fiber properties. Therefore, in the case of LC spinning, it is necessary to determine the concentration that produces the nematic phase.

The wet‐spinning process consists of three main unit operations in a complex continuous system: flow in a nozzle, drawing, and coagulation. Alignment, which is a critical factor in fiber properties, can be achieved by flow fields such as shear flow along the side wall in the nozzle and extensional flow by drawing (Figure 1c,d).^[^ ^12^, ^42^ ^]^ Extensional flow is more effective than shear flow for particle alignment.^[^ ^12^, ^43^, ^44^ ^]^ In our previous study, it was found that extensional deformation by drawing in the spinning process is the dominant process for the orientation of CNT fibers.^[^ ^12^ ^]^ In this work, the wet spinning process was performed for optimal conditions while controlling the draw ratio based on the HSL model by Lee.^[^ ^42^ ^]^ The CNT and G‐CNT fibers were obtained from a fully nematic phase (0.7 vol%) at an optimal draw ratio of 2.2 or higher (Figure S5, Supporting Information). Large‐scale continuous wet spinning through nozzles with up to 40 holes was performed, and the diameter of the fibers was controlled by the number and diameter of holes in the nozzles. In this study, all analyses of hybrid fibers were evaluated with single filaments of 10–12 µm.

### 2.2 Macroscopic Properties

**Figure 2:**
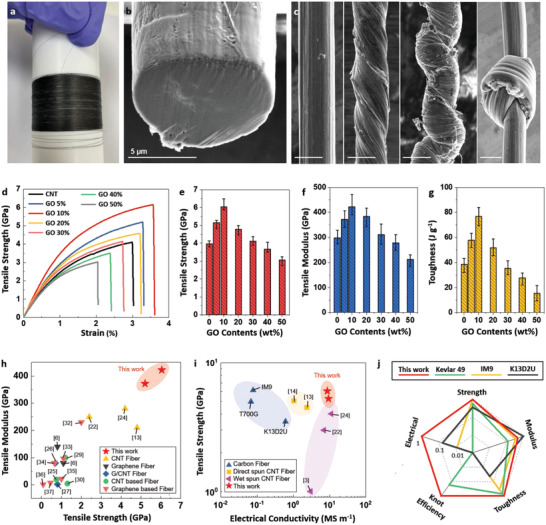
CNT and G‐CNT fibers and their macroscopic properties. a) G‐CNT fiber with diameter of 80 µm spun by 40‐hole nozzle. SEM images of b) cross section of G‐CNT fiber (single filament) and c) twisted and knotted G‐CNT fibers (single filament). The scale bar is 10 µm. d) Stress–strain curves, e) tensile strength, f) modulus, g) toughness of CNT and G‐CNT fibers. h) Comparison of tensile strength as a function of tensile modulus with CNT‐, graphene‐based composite, and hybrid fibers. i) Comparison of electrical conductivity as a function of tensile strength for CFs and CNT fibers. j) Radar chart comparing fiber properties required for multifunctional applications. The properties were normalized to the highest value among the fibers.

The outstanding flexibility of G‐CNT fibers, which can be twisted and knotted, makes them stand out compared to high‐performance CFs (**Figure** **2**a–c). The knot strength efficiency of G‐CNT fibers with 10% GO content was 92± 7%, which indicates flexibility far superior to that of polymeric fibers such as Kevlar 49 (Figure S6, Supporting Information).^[^ ^5^ ^]^ In addition, G‐CNT fibers exhibit excellent mechanical and electrical properties. The tensile strength, modulus, and toughness of the G‐CNT fibers increase dramatically with increasing GO content up to 10% and decrease more gradually up to GO contents greater than 20% (Figure 2d–g and **Table** **1**). At a GO content of 10%, the maximum tensile strength and modulus were 6.05± 0.45 GPa and 422± 49 GPa, respectively—increases of 53% and 42%, respectively, over those of CNT fibers. These results demonstrate that G‐CNT fibers exhibit tensile strength and modulus properties that are far superior to those of various hybrid and composite fibers, including CNT‐ and graphene‐based fibers (Figure 2h). Compared to IM9, which is a high‐performance CF with a tensile strength of 6.14 GPa and a modulus of 304 GPa, the tensile strength is similar, and the modulus is 39% higher. In addition, the electrical conductivity is 14 to 108 times higher than that of other CFs (Figure 2i). Compared with copper, a widely used conductive metal, the density is ≈4.5 times lower. In addition, the specific electrical conductivity is 65–83% level of copper, which suggests promise for use as an ultralight conductive wire (Table S1, Supporting Information). The G‐CNT fibers simultaneously exhibited excellent strength, modulus, toughness, knot efficiency, and electrical conductivity compared to CFs and Kevlar 49 (Figure 2j). Notably, the G‐CNT fibers prepared by wet spinning have outstanding performance characteristics, despite not requiring high‐temperature heat treatment processing, as is the case for graphene‐based fibers and PAN or pitch‐based CFs. Moreover, high‐performance CFs are mainly brittle, whereas G‐CNT fibers can be knotted, twisted, and manufactured as fabric (Figure 2c and Figure S7, Supporting Information). All of the excellent mechanical and electrical properties required for multifunctional applications, including flexibility, can be satisfied by G‐CNT hybrid fibers (Figure 2j).

**Table 1:** Tensile strength, modulus, toughness, electrical conductivity, knot strength efficiency, and specific density of CNT and G‐CNT fibers

GO contents [%]	0	5	10	20	30	40	50
Tensile strength [GPa]	3.96 ± 0.17	5.27 ± 0.34	6.05 ± 0.41	4.80 ± 0.38	4.17 ± 0.25	3.71 ± 0.38	3.06 ± 0.32
Tensile modulus [GPa]	297 ± 31	376 ± 35	422 ± 49	384 ± 45	312 ± 38	278 ± 33	210 ± 28
Toughness [J g^–1^]	38.4 ± 6.2	57.9 ± 5.8	76.8 ± 6.3	51.8 ± 6.9	35.3 ± 5.1	27.75 ± 5.5	15.5 ± 6.1
Knot strength efficiency [%]	85.3 ± 11.2	86.4 10.1	92.0 ± 7.2	93.2 ± 6.8	92.7 ± 6.2	92.6 ± 6.4	91.1 ± 8.1
Electrical conductivity [MS m^–1^]	10.4 ± 0.88	9.41 ± 0.81	8.43 ± 0.95	7.45 ± 0.91	5.61 ± 0.73	4.90 ± 0.52	4.28 ± 0.55
Specific density [g cm^–3^]	1.93 ± 0.02	1.98 ± 0.03	2.01 ± 0.02	1.99 ± 0.03	1.98 ± 0.03	1.96 ± 0.03	1.96 ± 0.03

### 2.3 Microstructures

**Figure 3:**
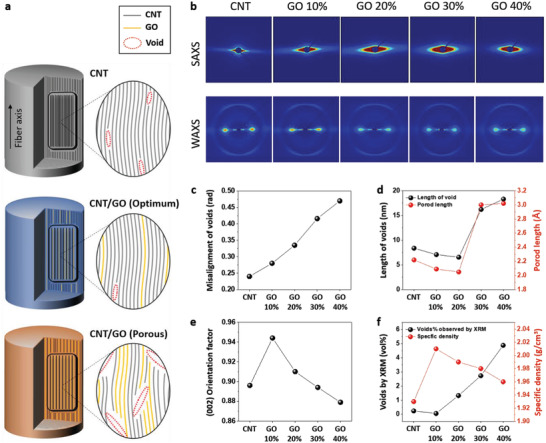
Microstructures of CNT and G‐CNT fibers. a) Schematic illustration of microstructures of CNT and G‐CNT fibers. b) 2D SAXS and WAXS pattern images of CNT and G‐CNT fibers. c) Misalignment angles of voids along the fiber axis. d) Length and Porod length of voids. e) CNT orientation factor along the fiber axis. f) Volume % of internal voids observed by XRM and specific density of the fibers.

**Figure 4:**
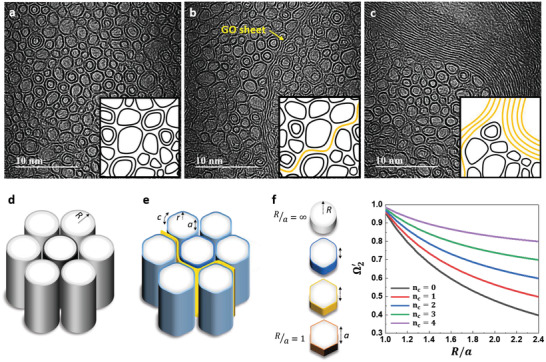
Microstructures of cross sections of CNT and G‐CNT fibers. HR‐TEM images of cross sections for a) CNT fibers, b) G‐CNT fibers with 10% GO content, and c) G‐CNT fibers with 30% GO content. Schematic illustration of bundles of d) circular CNTs and e) polygonised CNTs introduced in GO layer. f) *R*/*a* and *n*_c_ dependence of Ω_2_.

The GO content plays a critical role in the formation of fibers, and the fiber microstructure determines GO's tensile properties. Small angle X‐ray scattering (SAXS) and wide angle X‐ray scattering (WAXS) analyses were conducted to investigate the microstructure of the fibers (**Figure** **3**). A schematic illustration of the expected microstructure of GO and CNT in fiber is shown in Figure 3a. The microvoid structure changes are shown in the 2D SAXS and WAXS pattern images (Figure 3b). The sharp and fan‐shaped equatorial streaks in the SAXS patterns are increasingly converted to oval shapes with round edges with increasing GO content in the fibers, suggesting that misalignment of voids and distribution changes are generated. The misalignment and size of voids are calculated using Ruland's method and classical Porod's law, with fractal theory considered (Figure 3c,d).^[^ ^45^, ^46^ ^]^ By inserting 2D GOs into well‐dispersed 1D CNTs, voids oriented in a direction normal to that of the fiber axis can be formed because of the edges of the GO. The increase in the degree of misalignment of voids with increasing GO content is consistent with the findings of a previous study.^[^ ^6^ ^]^ This leads to the generation of a void distribution not along the fiber axis but rather normal to the direction of the fiber axis. Hence, misalignment angles of voids increase from 0.24 radians to 0.47 radians with increasing GO content. The void length and thickness (Porod length) were also observed to vary with the GO content. In addition to the misalignment of voids along the fiber axis, the void size tended to increase at GO contents above 30% (Figure 3d). The void length and thickness increased from 6.55 and 0.21 nm to 18.3 and 0.3 nm, respectively. When the cross sections of fibers were observed with high resolution transmission electron microscopy (HR‐TEM), monolayer GO was observed between CNTs at GO contents less than 20%, but as the GO content increased, multilayer GO sheets were observed between CNT bundles (**Figure** **4**a–c, and Figure S8, Supporting Information). In addition, the CNT bundles and multilayer GOs were finely separated on a scale of several tens to hundreds of nanometers (Figure S8, Supporting Information). The larger lateral size and higher concentration of GO in LC solution can induce the restacking of GOs.^[^ ^21^, ^47^ ^]^ A multilayer GO with high bending stiffness hinder the formation of an ideal structure in the equatorial direction in the fiber compared to a flexible GO monolayer. These graphitic layers induce the formation of a large number of pores with various sizes between themselves or CNT bundles.

The streak length in the 2D SAXS patterns increases slightly up to a 20% GO content and decreases at higher GO contents. The void length distribution decreased at a 40% GO content because of the formation of enlarged voids, which converted the fan‐shape of the streak to an oval shape. Similar with void length, there is a similar trend for void thickness (Porod length) resulted from an electron density difference^[^ ^48^ ^]^. Therefore, dramatically larger voids with long length and thickness are formed when the GO content is above 30%. These larger voids affect the misalignment of the (002) plane calculated from WAXS at 2*θ* = 26°. The orientation factor of the (002) plane of the G‐CNT fiber increased to 0.944 when containing 10% GO content (Figure 3e) and then decreased with increasing GO content in the G‐CNT fiber, as shown in Figure 3b–e and Figure S9 (Supporting Information). The (002) plane of the G‐CNT fiber with 20% GO content was misaligned despite its similar size of the voids with G‐CNT fiber containing 10% GO content. Combining these results with those from the X‐ray microscopy (XRM) analysis (Figure 3f) suggests that smaller but more numerous voids are generated in G‐CNT fiber containing 20% GO, affecting both the misalignment of the (002) plane and the tensile properties. It can be concluded that the tensile properties of the fiber are influenced by the void size and orientation factor of the (002) plane.

Fiber voids larger than several hundred nanometers can be quantified by XRM. In addition to the void information obtained from SAXS, internal voids quantified by XRM also reflect the mechanical properties of fibers. Compared to CNT fibers, the internal voids of G‐CNT fibers with 10% GO content were reduced by a factor of approximately eight to 0.03 vol% (Figure 3f and Figure S10, Supporting Information). In contrast, at GO contents above 20%, internal voids increase remarkably (Figure 3f). Despite the local observation of XRM, the porosity inside the fiber was in good agreement with the measured specific density (Figure 3f). The density of CNT depends on its diameter and the number of walls.^[^ ^49^ ^]^ The theoretical density of CNT used in this study was calculated to be 1.81 g cm^–3^ (Figure S11, details are provided in the Supporting Information), whereas the measured density of CNT fiber was 1.93 g cm^–3^. As the CSA can be spontaneously filled inside the CNT wall,^[^ ^50^ ^]^ the density can be increased because of the effect of the remaining CSA, despite the solidification process.^[^ ^12^, ^24^ ^]^ In practice, the doped CSA in the CNTs could be removed by heat treatment at 1400 °C or higher, at which time the density and electrical conductivity gradually decrease.^[^ ^15^ ^]^ In the case of G‐CNT fibers, the graphitic layers can also contribute to the density increase.

The increase in the tensile strength of G‐CNT fibers can be also explained by applying the simple fracture model for CNT fibers proposed by Vilatela et al.^[^ ^19^ ^]^ The specific stress of CNT fibers in the model is given by the following equation:

(1) $$\sigma^{\prime }=\frac{1}{6}\;\Omega_{1}\Omega_{2}\tau_{F}L$$

where σ' is the specific stress, *τ*_*F*_ is the interfacial fracture strength in shear, *L* is the mean length of CNT, Ω_1_ is the fraction of the total number of graphene layers that are on the outside of the CNTs and Ω_2_ is the fraction of the outer layer surface in contact with neighboring nanotubes, given by^[^ ^19^ ^]^

(2) $$\Omega_{2}=\frac{6a}{2\pi R}\;=\;1-\frac{r}{R}\;$$

where *a* is one of the flat sections of the nanotube and *R* is the radius of the equivalent perfectly circular tube (Figure 4d,e). If there are only circular CNTs, it can be considered that there is only line contact between adjacent tubes (Figure 4d). However, in practice, nanotubes with relatively large diameters inside the dense CNT fiber are flattened against each other, and a polygonal structure is formed, despite the increase in curvature energy required obtain the interactive area between tubes (Figure 4a) ^[^ ^19^ ^]^. In the case of G‐CNT fibers with GO contents less than 10%, monolayer GO was effectively introduced between nanotubes, and flexible GO layers were well distributed along the periphery of the circular CNTs (Figure 4b and Figure S8, Supporting Information). Monolayer GO sheets were formed tortuously along the CNT shape and eventually led to improvement in the bonding area. This is the factor that most straightforwardly affects Ω_2_, similar to the collapsed CNT.^[^ ^19^ ^]^ When monolayer graphene is embedded inside the CNT bundle, Equation (2) can be modified as follows

(3) $$\Omega_{2}^{\prime }=\frac{6a+cn_{c}}{2\pi R}\;$$

where *c* is one of the curved sections of the polygonal tube and *n_c_* is the value of *c* corresponding to the number of monolayer graphene contacts in a nanotube (Figure 4e). If the nanotube is a regular polygon with no curves, then *R*/*a* is 1, and as *R*/*a* increases, it becomes closer to a circle. In the case of collapsed CNT, it can be *R*<*a*.^[^ ^19^ ^]^ The introduction of a flexible graphene sheet into the bundle enables the utilization of the *c* region of the nanotube, and the increase in *n_c_* also increases the $\Omega_{2}^{\prime }$ (Figure 4f). Although it is difficult to be precise, the *n_c_* was observed to be ≈1–2 in the HR–TEM image (Figure 4b). At *R*/*a* = 2.0, when the values of *n_c_* are 1 and 2, the values of $\Omega_{2}^{\prime }$ are 0.56 and 0.65, which are ≈18% and 36% greater, respectively, than without a graphene layer (Figure 4f). However, an increase in the contact area cannot be expected for all nanotubes because of the limited monolayer GO content inside the fiber. Additionally, the oxygen functional groups of GOs may create a greater frictional force between CNT and GO than smooth CNT surface, leading to an increase in the *τ*_*F*_. Indeed, GOs are known to create a larger frictional force than graphene.^[^ ^31^, ^51^ ^]^

Even if the lateral size of GO is different, the tensile strength of the G‐CNT fibers exhibits the same trends (Figure S12, Supporting Information). However, the maximum tensile strength increases as the lateral size of the GOs decreases (Figure 2e and Figure S12, Supporting Information). When the content of large GOs with an average lateral size of 37 µm is 15% or more, even fiber production by drawing is impossible. Since the exfoliation of large GOs (>30 µm) may be more difficult, multilayered large sheets can further induce the development of graphitic structures inside the fibers, creating more internal voids. Conversely, intercalated smaller GOs between nanotubes may lead to effective packing of the hybrid fibers.^[^ ^31^ ^]^ Similarly, the optimal combination of small and large GOs can form a compact graphene fiber structure.^[^ ^6^ ^]^ In multidimensional assemblies of CNT and GO, the components’ content ratio and dimensions significantly affect the microstructures and macroscopic properties.

### 2.4 Surface and Electrochemical Properties

**Figure 5:**
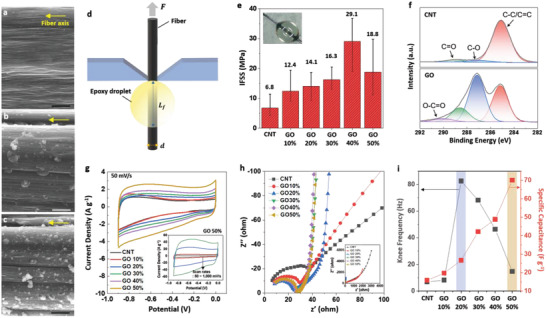
Surface properties and electrochemical analysis of CNT and G‐CNT fibers. SEM images of a) CNT fiber, b) G‐CNT fiber with 20% GO content, c) G‐CNT fiber with 40% GO content. The scale bar is 1 µm. d) Schematic illustration of IFSS measurement. e) IFSS of CNT and G‐CNT fibers. f) XPS spectra of CNT and GO. g) CV profiles for the CNT and G‐CNT fiber series (inset: CV profiles of G‐CNT fibers with 50% GO content at scan rates from 50 to 1000 mV s^−1^). h) Nyquist plot of CNT and G‐CNT fiber series in the high‐frequency region (inset: Nyquist plot in the low‐frequency region). i) Left: knee frequency; right: specific capacitance of CNT and G‐CNT fiber series.

As the GO content increases, the surface roughness of the G‐CNT fibers is enhanced because the amount of GO aggregates protruding from the G‐CNT fiber surface increases (**Figure** **5**a–‍c). In fiber‐reinforced polymer composites (FRPs), the interfacial shear strength (IFSS) between the fibers and matrix resin is decisive in determining the mechanical properties.^[^ ^52^ ^]^ The IFSS of the G‐CNT fibers was measured by microdroplet testing using epoxy, which is the most widely used resin for FRP (Figure 5d,e). Compared to CNT fibers, with a smooth and clean surface (Figure 5a), the IFSS was increased by 1.82 times even with the addition of a small amount of 10% GO. Moreover, the IFSS of the G‐CNT fibers with 40% GO contents increased by a factor of 4.3, peaking at 29.1 MPa. However, at a 50% GO content, the IFSS decreased because of the fiber exfoliation at the outermost layer of the bundle, not because of interfacial failure with epoxy resin (Figure S13, Supporting Information). The rough surface formed by the GO increases the specific surface area and increases the mechanical interlocking with a matrix resin in the interface.^[^ ^53^, ^54^ ^]^ Furthermore, the oxygen‐containing functional groups (C—O and C=O) of the GO on the surface of the G‐CNT fibers form hydrogen bonds with the epoxy matrix, which facilitates load transfer and improves the IFSS (Figure 5f and Figure S14, Supporting Information).^[^ ^55^, ^56^ ^]^ Therefore, the G‐CNT fibers by addition of GOs can have both mechanical surface area increase and chemical sizing effect of CFs without any additional processing.

The electrochemical performance of G‐CNT fibers with different amounts of GO was evaluated in a half‐cell using a single fiber. A CNT fiber exhibits a nearly rectangular cyclic voltammetry (CV) curve shape, illustrating its excellent electrical conductivity. G‐CNT fibers exhibit larger CV curves than pure CNT fiber due to the effect of both surface modification and open‐microstructure by incorporated GO (Figure 5g, Figures S10 and S14, Supporting Information). The total area under the CV curve of the G‐CNT fiber increases gradually as the amount of GO incorporated is increased, indicating enhanced volume of the void and surface functionality for adsorption of ions (Figure 3f, Figures S10 and S14, Supporting Information). In particular, G‐CNT fiber with a 20% GO content exhibits a sharp response at the voltage changing point, even at a high scanning rate of 1000 mV s^–1^ (Figure S15, Supporting Information), representing the fast charge/discharge characteristics originating from the high conductivity and favorable surface characteristics. Although the best‐performing G‐CNT fiber with 50% GO content, exhibited a slightly distorted CV curve compared to the G‐CNT fiber with a 20% GO content, it also maintained a rectangularly shaped CV curve at a fast scan rate of 1000 mV s^–1^, exhibiting the highest specific capacitance of 70 F g^–1^ at the current density of 1 A g^–1^ (Figure 5g inset, Figure 5i, and Figure S16, Supporting Information). The best performed G‐CNTF with 50% GO content exhibits a noticeably higher electrochemical performance than other similar CNT/GO composite fibers such as GO@CNT fiber (64 F g^–1^ at 1 A g^–1^)^[^ ^57^ ^]^, reduced GO/unzipped CNT fiber (53.9 F g^–1^ at 0.1 A g^–1^)^[^ ^58^ ^]^, G/CNT fiber (50 F g^–1^ at 50 mV s^–1^)^[^ ^59^ ^]^, and GO/CNT/Cotton fiber (1.25 F g^–1^ at 2 mV s^–1^)^[^ ^60^ ^]^ (Figure S17, Supporting Information). Electrochemical impedance spectroscopy (EIS) analysis can provide further information on the kinetic properties of composite materials. Figure 5h shows a typical Nyquist plot for CNT and G‐CNT fibers in the frequency range of 100 kHz to 0.05 Hz. In the high‐frequency region, G‐CNT fibers exhibit reduced charge transfer resistance (*R*_ct_) compared to pure CNT fiber, demonstrating again the advantageous properties achieved by the addition of GO to CNT fiber (Figure 5h). In the low‐frequency region, the vertical feature of the Warburg impedance exhibits the nearly ideal capacitive behavior of the G‐CNT fiber (Figure 5h inset). For the Warburg impedance, the knee frequency is defined as the maximum frequency below which predominantly capacitive behavior can be maintained, so a high knee frequency is desirable for the electrode materials of a supercapacitor.^[^ ^61^ ^]^ The knee frequencies extracted from the Nyquist plot are summarized in Figure 5i. Interestingly, G‐CNT fiber with a 20% GO content exhibits the highest knee frequency (82 Hz) and the smallest relaxation time (47 ms) among the samples tested, which means that G‐CNT fiber with GO 20% has the most optimized structural characteristics for fast charge/discharge operations (Figure 5i and Figure S18, Supporting Information). It should be emphasized that even though G‐CNT fiber with 50% GO exhibited the highest specific capacitance because of sufficient volume of the void surface functionalities, there might be a trade‐off between electrochemical performance and electrical conductivity. Therefore, it can be concluded that the structural and electrochemical properties of G‐CNT fiber are tunable and can be specifically customized by changing the ratio of CNT to GO.

## 3 Conclusion

This paper presented a simple and powerful method for assembling high‐performance fibers that are hybrids of CNT and GO. LC spinning with CSA facilitates the dispersion of CNT and GO, the alignment and packing of fibers, and the creation of a unique multidimensional structure. The microstructure of the fibers was a function of the physical dimensions and content of 2D GO. The ratio of the two distinct materials used, CNT and GO, was found to dramatically affect the macroscopic properties of the hybrid fiber by improving the fiber orientation, reducing voids, and maximizing interfacial contact. The optimal ratio of the two components depends on their physical dimensions. The G‐CNT hybrid fiber exhibits mechanical and electrical properties far superior to those of previously reported CNT‐ and graphene‐based hybrid and composite fibers. In addition, the mechanical properties of the hybrid fiber are competitive with those of traditional CFs, and their electrical properties and flexibility are far superior. The G‐CNT fibers assembled by the process described in this paper exhibit significant advantages for commercialization, as they do not require high‐temperature heat treatment, which is commonly required for CFs. In addition, the improved surface and electrochemical properties of the G‐CNT fibers are tunable, depending on the content of GO. These superior properties can be achieved for individual fibers, and their unique combination can be useful for multifunctional applications, such as wearable electronics, robotics, composites, and lightweight wires.

## 4 Experimental Section

### Materials

CNT and GOs were prepared from Meijo Nano Carbon (DX‐2) and Grapheneall, respectively. Chlorosulfonic acid (CSA) was purchased from Sigma–Aldrich. The acetone (99.5%) for coagulant was prepared from Daejung.

### Wet Spinning

The LC solutions (CNT/GO/CSA) were spun through 1‐, 5‐, 24‐, and 40‐hole nozzles (inner diameter of 0.15 mm). The LC solutions spun out of the spinneret were immediately solidified in acetone. The coagulated fibers were obtained using a winding drum. The flow rate (0.1–0.5 mL min^–1^) and draw ratio were controlled by a syringe pump (Fusion 710, Chemyx) and the winding rate, respectively. The wet‐spun fibers were washed with distilled water for 6 h and dried at 80 °C for 24 h in a vacuum oven.

### Mechanical Properties

The tensile properties and linear density (tex) were measured using a FAVIMAT+ (Textechno). Tensile tests were performed with fibers 25 mm long. The pre‐tension was 1–2 cN, and the strain rate was 2 mm min^–1^. The tensile modulus was calculated from the 0 to 0.3% strain range of the stress–strain curve. The linear density was measured by calculating the resonance frequency obtained with a vibroscope.^[^ ^62^ ^]^ The resonance frequency was measured while increasing the pre‐tension from 1 to 2 cN at a rate of 2 mm min^−1^. All tensile test specimens were measured at least 50 times.

### X‐Ray Diffraction

Small and wide‐angle X‐ray scattering (SAXS, WAXS) measurements were obtained using the PLS‐II 6D UNIST‐PAL beamline of the Pohang Accelerator Laboratory (PAL) in Korea to characterize the range of voids and the CNT orientation factor. 2D SAXS and WAXS pattern images were obtained by Rigaku Micromax‐007 (operated at 45 kV, 66 mA, *λ* = 0.154 nm) using a Rigaku *R*‐axis IV++ detection system with different sample‐to‐detector distances (SDDs). Each SAXS and WAXS SDD was ≈3060 and 250 mm.

### Characterization of Void Structure

The misalignment angles and void lengths along the fiber axis were calculated from an azimuthal SAXS scan using the Ruland streak method.^[^ ^45^ ^]^ A single cylindrical void shape along the fiber axis was scattered in an equatorial streak with the fiber axis. If voids are distributed with misalignment along the fiber axis, the fan‐shaped streak is presented in a 2D SAXS pattern. Thus, the orientation distribution of the microvoids along the fiber is enabled to be calculated from the azimuthal spread in the equatorial streak of the fiber. This azimuthal spread was characterized from the observed integral breadth (*B*_obs_) of the convolution in azimuthal angle (in radians) with a function of s in reciprocal space:

(4) $$B_{\mathrm{obs}}\;\left(s\right)=\;\frac{\int_{\varphi_{\max}-\frac{\pi }{2}}^{\varphi_{\max}+\frac{\pi }{2}}I\left(s,\varphi \right)\;d\varphi }{I\left(s,\varphi_{\max}\right)}$$

where *φ*_max_ is the angle at the intensity peak maximum. Intensity (*I*(*s*,*φ*)) in azimuthal scan is obtained at constant scattering vector *s* = 2 sin *θ*/*λ* where 2*θ* and *λ* represent scattering angle and wavelength of X‐ray respectively and *φ* is azimuthal angle measured from the scattered streak. A Gaussian–Lorentz distribution was applied to obtain orientation distribution and relation between misalignment angles of voids (*B*_g_) and length of the void (*L*_v_) with the following linear relation

(5) $$s^{2}\;B_{\mathrm{obs}}^{2}=\;\frac{1}{L_{v}^{2}}+s^{2}B_{g}^{2}$$

where *B*_g_ is the true integral breadth of the orientation distribution, showing the microvoids along the fiber axis. *L* and *B*_g_ are calculated from the slope of the plot.

### Calculation of Porod Length (*l*_p_)

In the Porod region (scattering vector *s*, s ≈ 1 nm^–1^), the slope of ln (*I*(*s*)) ≈ ln(*s*) represents the surface of the void and it determines Porod length where *s* and *I*(*s*) represent scattering vector and intensity with direction of equatorial direction. When the value of slope is under 4 as shown in Figure S19 (Supporting Information), the material represents a smooth interface, therefore, classical theory is applied to the calculation of void thickness as called chord length or Porod length with consideration of fractals.^[^ ^46^ ^]^

(6) $$l_{p}=\frac{4\pi \int s^{2}I\left(s\right)ds}{2\pi^{3}\lim_{s\to \infty }s^{4}I\left(s\right)}\;$$

### Orientation Factor of (002) Plane

Carbon_(002)_ orientation factor was calculated by Herman's orientation factor^[^ ^63^ ^]^ through azimuthal scan of the diffraction peak at 2*θ* ≈ 26° with applying Gaussian distribution function.

(7) $${\mathrm{Herman}}^{\prime }s\;\mathrm{orientation}\;\mathrm{factor},\;P_{2}\;\left(\cos\beta \right)=\frac{\left(3\cos^{2}\beta -1\right)}{2}\;$$

where is *β* the angle between crystal plane and fiber axis.

### Interfacial Shear Strength

The IFSS between the CNT fiber and epoxy resin was measured by a single fiber microdroplet debonding test and calculated by the following Equation (1)

(8) $$\tau \;=F/\pi dL_{f}\;$$

where *τ* is the IFSS, *F* is the maximum load, *d* is the fiber diameter and *L*_f_ is the embedded length of the resin.^[^ ^64^ ^]^ Micro droplets were prepared by mixing an epoxy resin (YD‐128, Kukdo) and a curing agent (MDA‐60, Kukdo) in a weight ratio of 10:3. The diameter of the CNT fibers was 20 µm and the embedded length by the micro droplet was controlled to 200–600 µm. A CNT fiber was pulled out with 0.5 mm min^–1^ speed and at least 25 measurements were averaged for each IFSS value.

### Rheological Measurements

The LC phase was observed with an optical microscope with a cross‐polarizing filter (L150, Nikon) by injecting an LC solution between two glass substrates of controlled thickness. The steady shear viscosity of LC solutions was measured using the PTFE tubes by employing the standard capillary rheometer technique at a shear rate of 52 s^–1^.^[^ ^42^ ^]^

### Electrochemical Measurement

The electrochemical properties of CNTF and G‐CNTF wear measured by a CHI 920D in aqueous electrolyte of 1 m KOH. The reference and counter electrodes were a saturated Ag/AgCl (3 m KCl) and Pt mesh, respectively. A single fiber of 5.5 cm was used as the working electrode. The electrochemical characteristics were investigated using cyclic voltammetry (CV, scan rate 50 ≤ *v* ≤ 1000 mV s^–1^), Galvanostatic charge/discharge (GCD, 1 A g^–1^) and electrochemical impedance spectroscopy (EIS, frequency range from 1 to 1 MHz, amplitude of 10 mV). The specific capacitance was calculated from the GCD profiles except for the IR drop at diverse current densities by the following equation:

(9) $$\mathrm{Capacitance}\;=\frac{I\times \Delta t}{m\times \Delta V}\;\;\left({F\;g}^{-1}\right)$$

where *m* is the mass of the fiber electrode (g), *I* is the discharge current (A), Δ*t* (s) is the discharge time, and Δ*V* (V) is the potential window.

### Characterizations

The cross‐sections of the fibers were prepared using a focused ion beam (FIB) of FEI‐Helios SEM. The microstructures of the cross‐sections of the fibers were observed by HR‐TEM (FEI‐Titan Cubed 60–300) and XRM (ZEISS Xradia 810 Ultra). The electrical conductivity was measured using the four‐point probe method with a probe station (MST‐4000A, MS Tech). The thermogravimetric analysis (TGA) was performed using a Q50 (TA Instruments) at a heating rate of 10 °C min^–1^ under an air atmosphere. The G/D ratios of CNTs, GOs, and G‐CNT fibers were measured by Raman spectroscopy (InVia Reflex, Renishaw) with 514 nm excitation wavelengths. Hybrid fibers’ oxidation state and carbon‐to‐oxygen atomic ratio were analyzed using an X‐ray photoelectron spectroscope (XPS, Thermo Scientific K‐Alpha).

### Statistics

For mechanical testing, specimens of the same length (25 mm) were measured at least 50 times. Electrical conductivity was evaluated at more than 10 points on the fibers. Linear density of fibers was measured more than 10 times on specimens of the same length (25 mm). Specific density of fibers was measured more than 5 specimens by density column apparatus. All the evaluated values were presented in form of mean ± standard deviation.

## Conflict of Interest

The authors declare no conflict of interest.

## Supporting information

Supporting Information

## Data Availability

The data that support the findings of this study are available in the supplementary material of this article.
