# Supplementary material for: Ultrastrong Hybrid Fibers with Tunable Macromolecular Interfaces of Graphene Oxide and Carbon Nanotube for Multifunctional Applications
Source: Adv Sci (Weinh). 2022 Aug 21;9(29):2203008. doi: 10.1002/advs.202203008 (PMC9561868; doi:10.1002/advs.202203008)
Supplement: Supplementary file 1 — Supporting Information [file ADVS-9-2203008-s001.pdf]

ADVANCED SCIENCE
Open Access

Supporting Information

for *Adv. Sci.*, DOI 10.1002/advs.202203008

Ultrastrong Hybrid Fibers with Tunable Macromolecular Interfaces of Graphene Oxide and Carbon Nanotube for Multifunctional Applications

*Seo Gyun Kim, So Jeong Heo, Jeong-Gil Kim, Sang One Kim, Dongju Lee, Minkook Kim, Nam Dong Kim, Dae-Yoon Kim, Jun Yeon Hwang, Han Gi Chae and Bon-Cheol Ku**

Supporting Information

Ultrastrong Hybrid Fibers with Tunable Macromolecular Interfaces of Graphene Oxide and Carbon Nanotube for Multifunctional Applications

*Seo Gyun Kim, So Jeong Heo, Jeong-Gil Kim, Sangyoun Kim, Dongju Lee, Minkook Kim, Nam Dong Kim, Dae-Yoon Kim, Jun Yeon Hwang, Han Gi Chae, and Bon-Cheol Ku**

Characterizations of CNTs and GOs

It is known that the purity, crystallinity (graphitic character), and length of CNTs are important in the manufacture and properties of CNT fibers.[1] We prepared the CNT from Meijo Nano Carbon (DX-2). Through the TGA curve of CNTs in air atmosphere, it can be confirmed that the content of impurities such as residual catalyst is 2.5% (Figure S1b). The G/D ratio of CNT (ratio of G peak and D peak) obtained Raman spectroscopy was 43, which can be considered to have sufficiently good crystallinity (Figure S1c). The carbon-to-oxygen atomic ratio of CNT by XPS analysis was 13.1 (Figure 4f).

The length (or aspect ratio) of CNT was estimated from the extensional viscosity (η^E) by capillary breakup extensional rheometer or dripping onto substrate rheometry.[2,3] The filament diameter of the CNT solution formed between tube and substrate decreases exponentially with time, while the η^E can be estimated from the mid-filament diameter (D_m)[2,4,5]

$$\eta^E = \frac{(2X-1)\sigma}{-\mathrm{d}D_\mathrm{mid}/\mathrm{d}t} \tag{S1}$$

where σ is the surface tension, and t is the time. The σ of CSA was 38 mN m^{-1},[2] and X= 0.7127, which is a dimensionless number for a viscous Newtonian fluid filament with a smoothly necked profile.[2,5] In addition, η^E can be estimated for a fully aligned semidilute solution and monodisperse rigid rods as follows[2,4,5]

$$\eta^E = \eta_s \left(3 + \frac{4\phi \left(\frac{L}{d}\right)^2}{3\left[\ln\left(\frac{1}{\phi}\right) + \ln\left(\ln\left(\frac{1}{\phi}\right)\right) + 0.1585\right]} \right) \tag{S2}$$

where η_s is the shear viscosity of solvent. The η_s of CSA is 0.0028 Pa·s.[2] The η^E of CNT/CSA solution (0.015 vol%) obtained from neck profiles was 1.80±0.21 Pa·s (Figure S2). When the average diameter of CNT is 1.6 nm, the average length of CNTs was estimated to be 9.6 μm.

The average lateral size of GOs was 1.72 μm (Figure S3a and b). The carbon-to-oxygen atomic ratio of GO was 1.7 (Figure 4f). The G/D ratio of GO obtained from Raman spectroscopy was 1.3 (Figure S3c).

Density of CNT

The density (ρ_{CNT}) of individual CNT depends on the number of walls (*n*) and diameter (*d*). The ρ_{CNT} can be estimated as following[6]

$$\rho_{CNT} = \frac{4000}{A_S(d + \delta_{\mathrm{vdW}})^2}\left[nd - 2\delta_{\mathrm{vdW}}\sum_{i=0}^{n} i\right] \tag{S3}$$

where $A_S = 1{,}315$ m^2 g^{-1} is the specific surface area for one side of a graphene sheet, and δ_{vdW} is the interlayer distance between two CNT walls. The density of SWNT, DWNT, and TWNT depending on the *d* is in Fig. S11a. From the HR-TEM images of cross-section of the CNT fiber, it was confirmed that the CNTs consist of a mixture of SWNTs, DWNTs, and TWNTs (Figure S11b). The *n* distribution and the *d* distribution of the CNT used in this work is in Fig. S11c-f. Herein, if there are only π-π interactions between CNTs, and the difference in diameter of CNTs is not large and the diameter of CNTs does not change, the density of perfectly aligned and packed CNT assemblies can be considered as the average density. From the distributions of diameter and wall number, the average density of CNTs used in this work was calculated to be 1.81 g cm^{-3}. However, it should be noted that the density may vary if distinct materials are encased inside the CNT walls, because the inside the CNT wall is considered as empty space in theoretical density.

Density measurement of fibers

The density of CNT and hybrid fibers was measured by a density gradient column of liquid (POLYTEST, Ray-Ran, UK). Two liquids (1,1,2,2,-tetrabromoethane and benzene) were used to bulid the density gradient, and glass beads with accurately known density floated in the column.[7] The fibers were inserted and left for 24 hr in the column. Furthermore, the density of fibers measured by the density column method was reconfirmed through the linear density (tex) obtained from FAVIMAT+ (Textechno) and the cross-sectional area obtained from SEM.

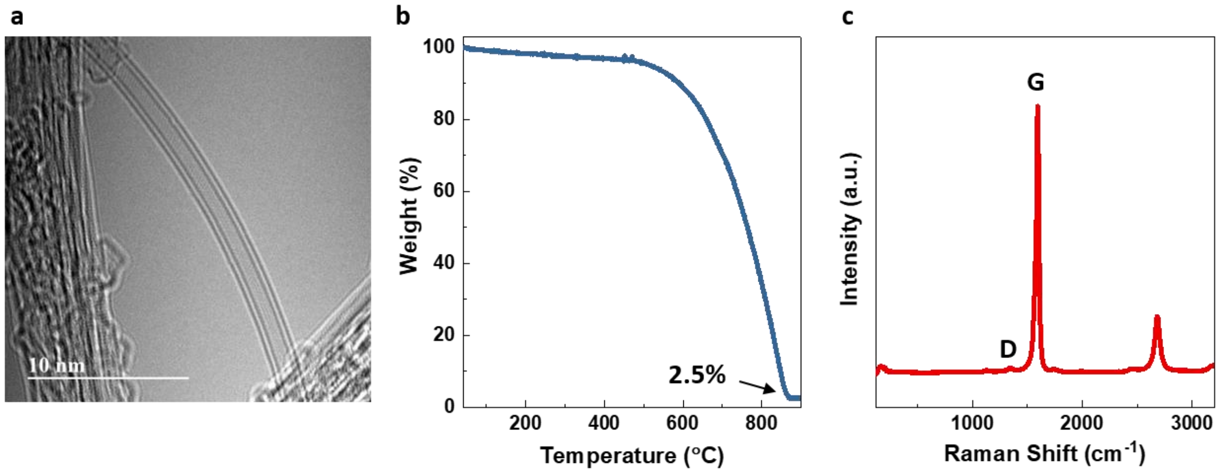

Figure S1. Characterizations of CNT (Meijo, DX-2). (a) TEM images of CNTs. (b) TGA curve of CNT in air. (c) Raman spectra of CNT.

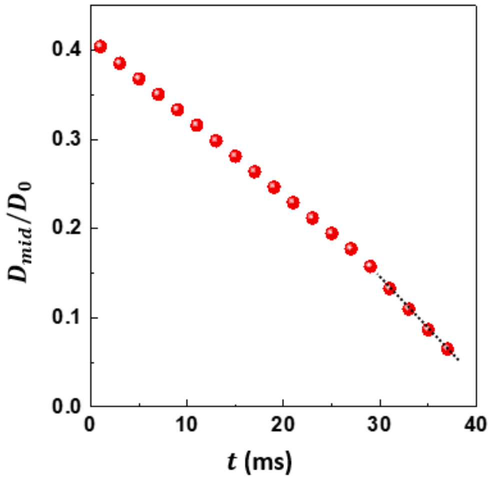

Figure S2. The diameter evolution with time of CNT solution (0.015 vol%) by the dripping-onto-substrate (DoS) rheometry for CNT aspect ratio.

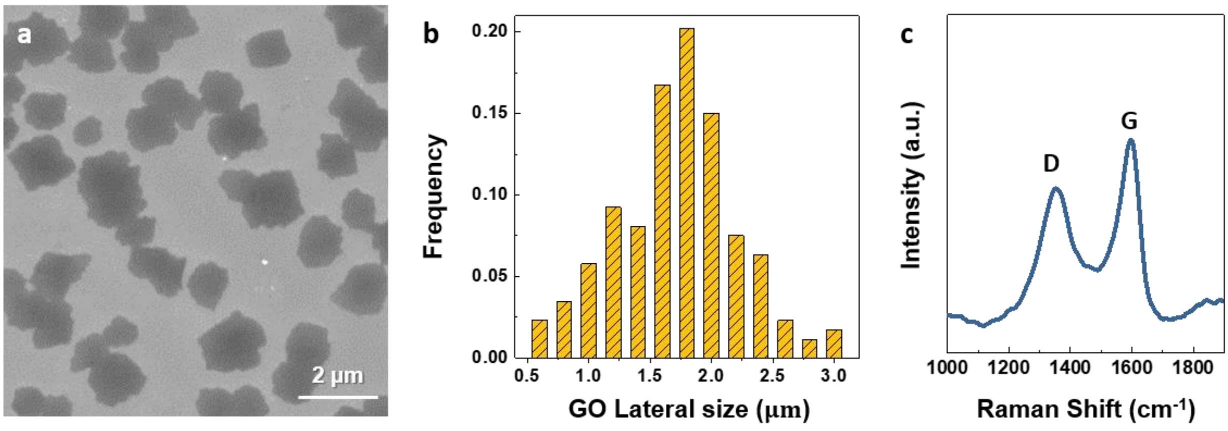

Figure S3. Characterizations of GO. (a) SEM image of GO sheets. (b) Distribution of lateral size of GOs, (c) Raman spectra of GO.

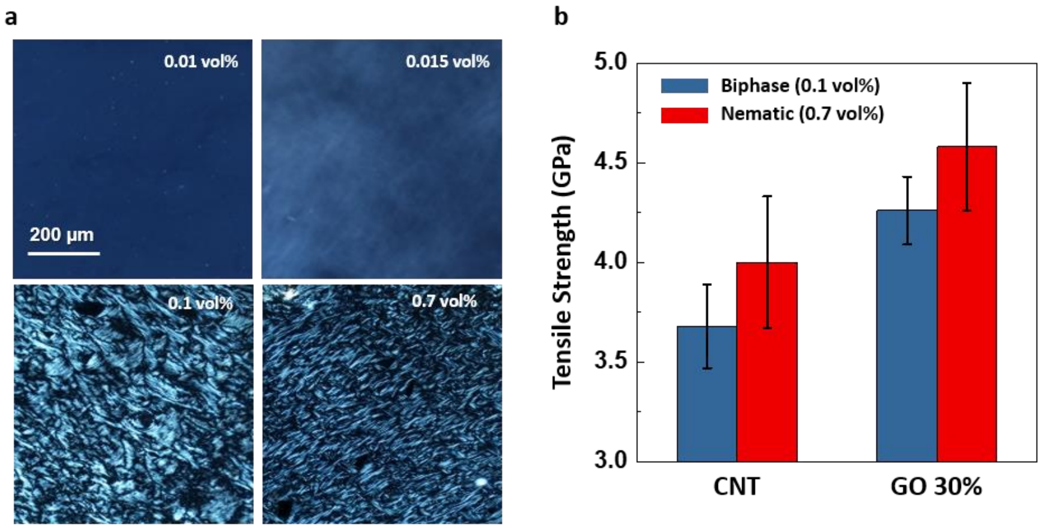

Figure S4. (a) Polarized optical images of CNT/GO (GO 10%) solutions. (b) Difference in tensile strength when spun under the same conditions (draw ratio of 2.4, spinneret diameter of 0.26 mm, flow rate of 0.1 ml min^{-1}) with biphase solution and nemaic phase solution.

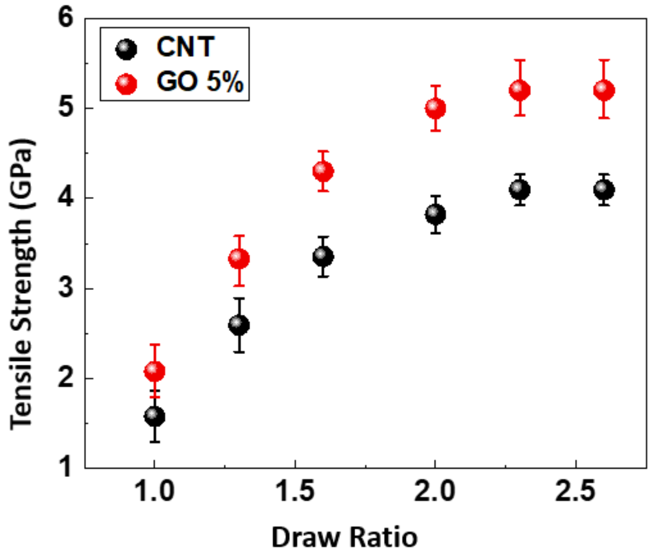

Figure S5. Tensile strength of CNT and G-CNT fibers depending on draw ratio for optimal processing conditions.

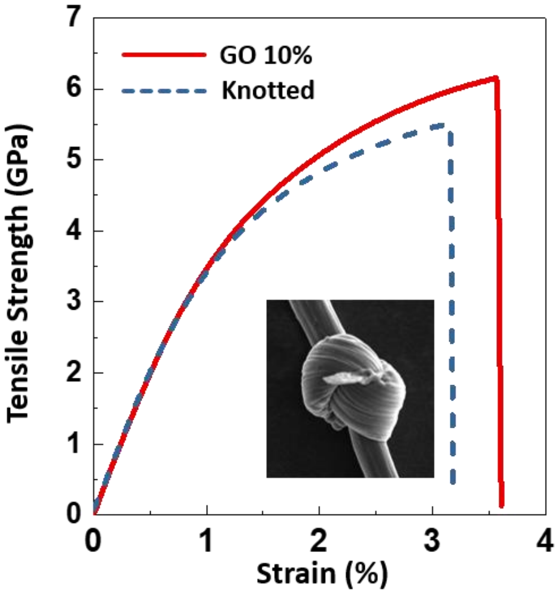

Figure S6. Knot strength efficiency of G-CNT fiber with 10% GO content.

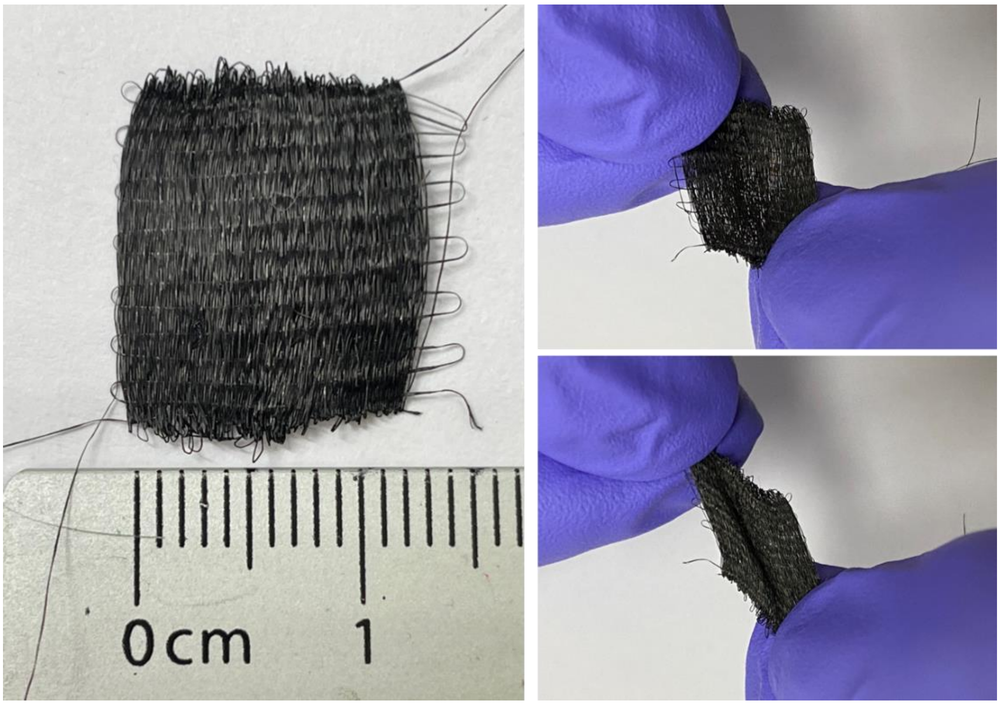

Figure S7. The 1.5 cm × 1.2 cm fabric manufactured by G-CNT fibers.

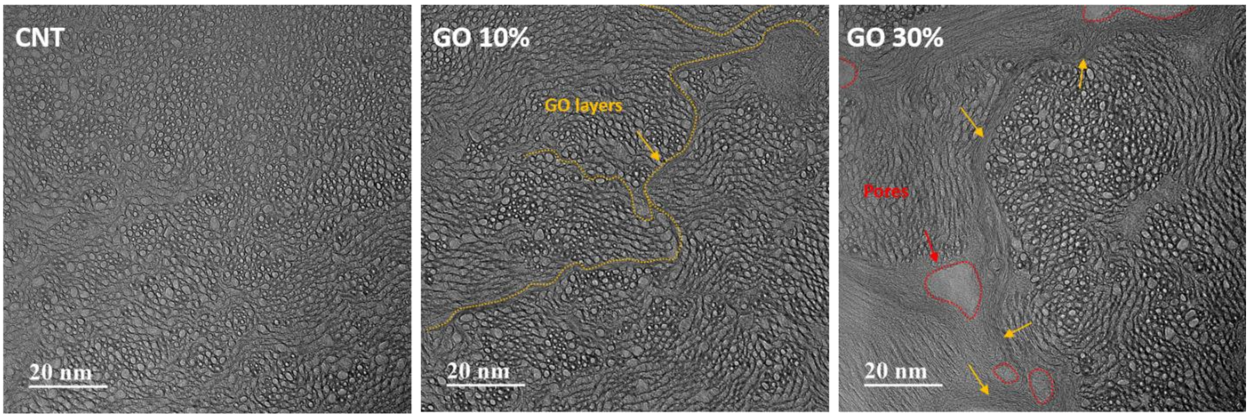

Figure S8. Cross-sections of G-CNT fibers observed by HR-TEM.

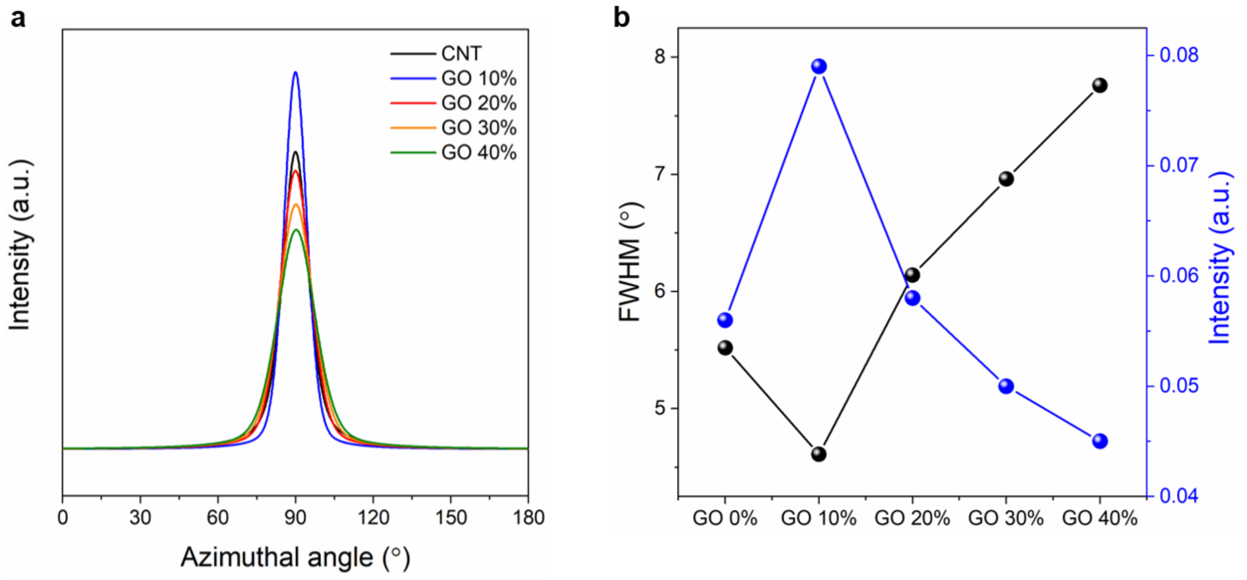

Figure S9. (a) Azimuthal plots obtained from 2D WAXS pattern image and (b) its each peak FWHM and intensity.

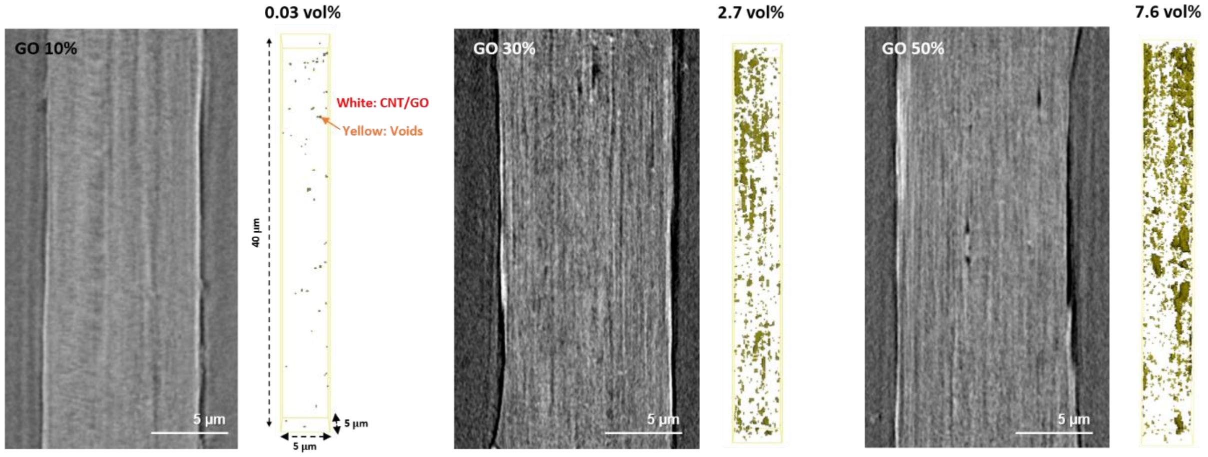

Figure S10. The internal voids of G-CNT fibers observed by XRM.

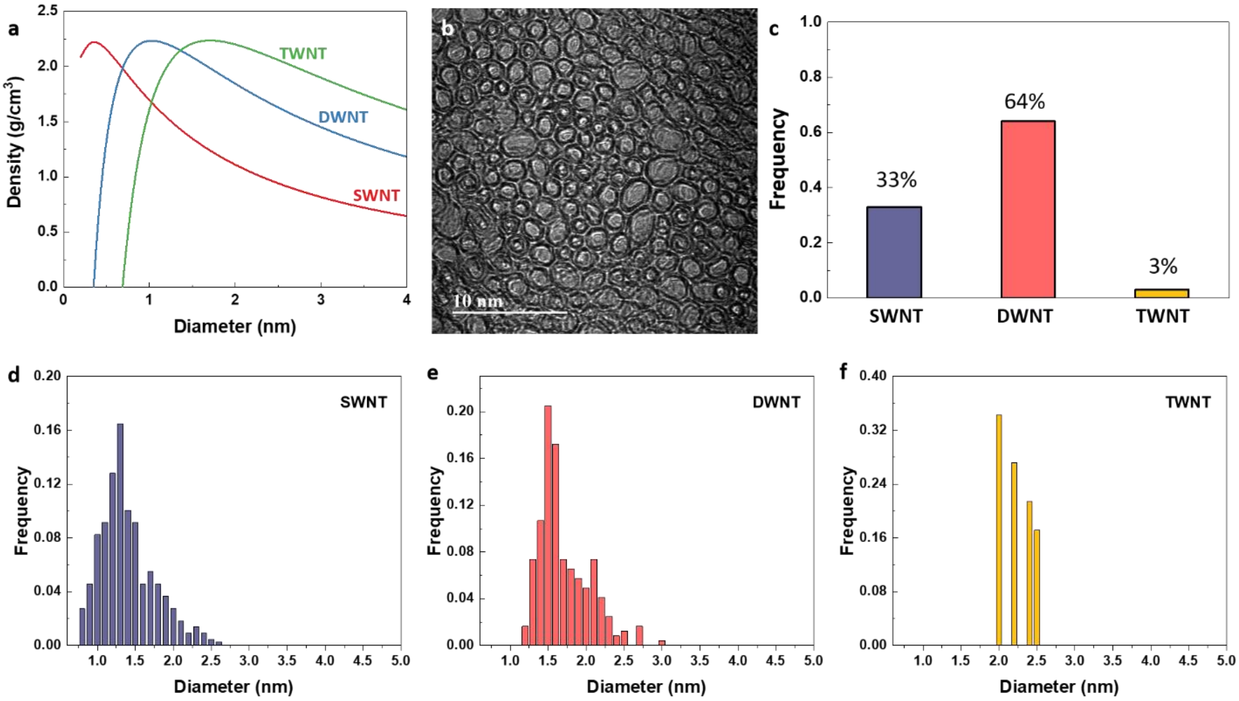

Figure S11. (a) Density of individual CNT depending on number and diameter of wall. (b) Cross-section of CNT fiber observed by HR-TEM. (c) Wall number distribution of CNT. (d) Distribution of wall diameter for SWNT. (e) Distribution of wall diameter for DWNT. (f) Distribution of wall diameter for TWNT.

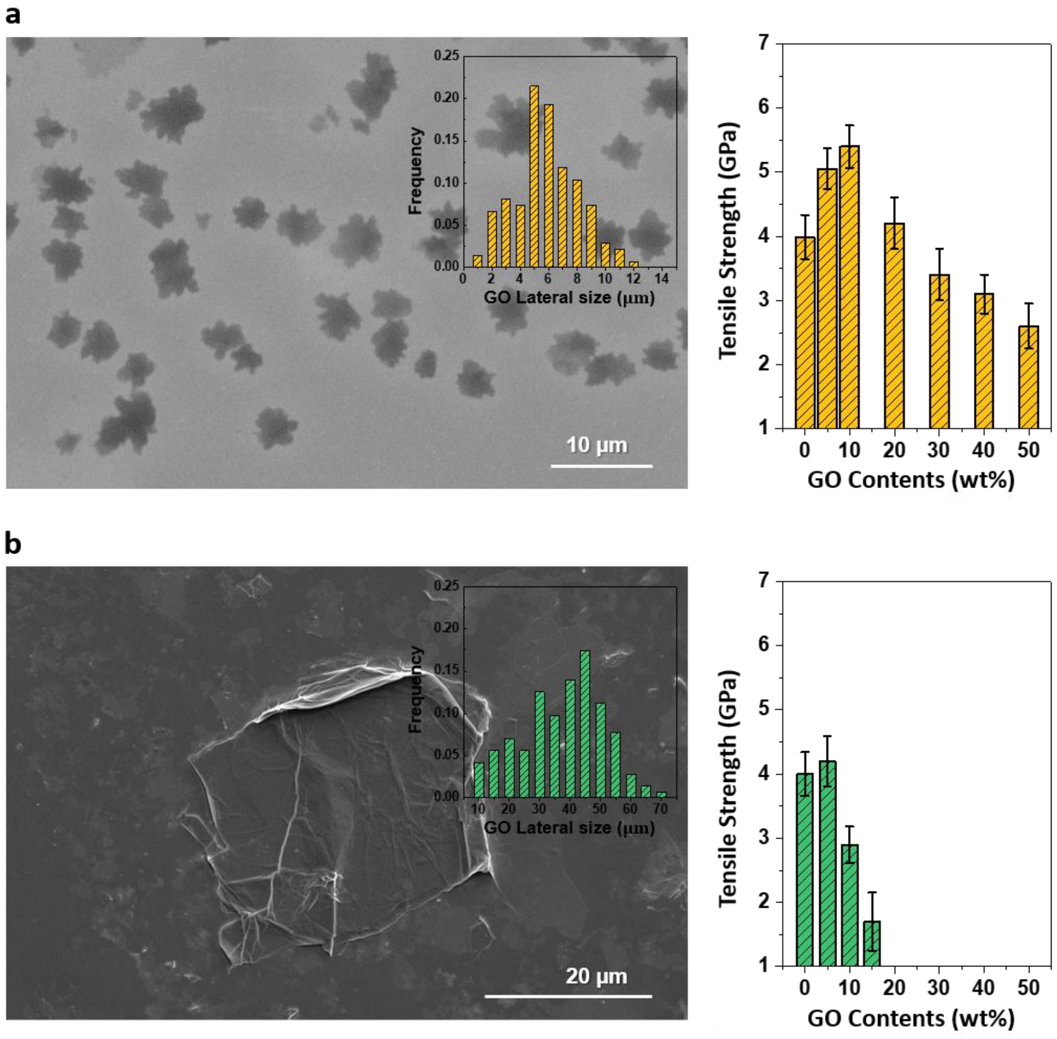

Figure S12. Tensile strength of G-CNT fibers depending on GO lateral size. Tensile strength of G/CNT fibers when the average lateral size of GOs is (a) 5.9 μm and (b) 37 μm, respectively.

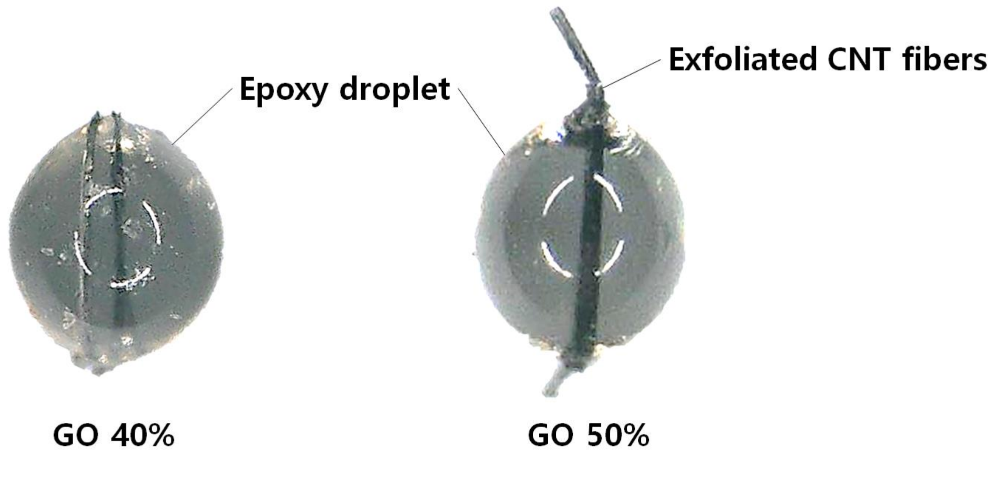

Figure S13. Epoxy Micro droplet after the pull out tests.

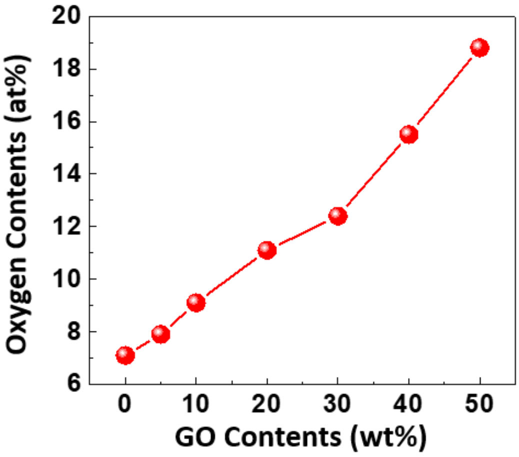

Figure S14. Oxygen contents of CNT and G-CNT fibers depending on GO contents.

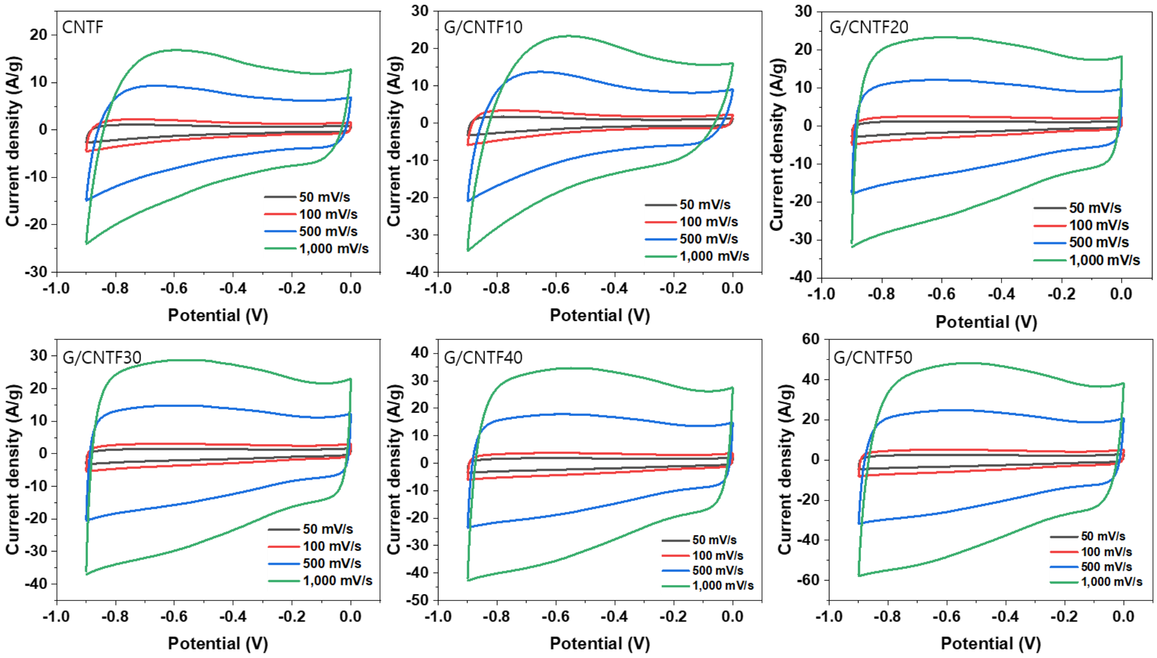

Figure S15. CV profiles for (a) CNTF, (b) G-CNTF10, (c) G-CNTF20, (d) G-CNTF30, (e) G-CNTF40, and (f) G-CNTF 50 at the diverse scan rates.

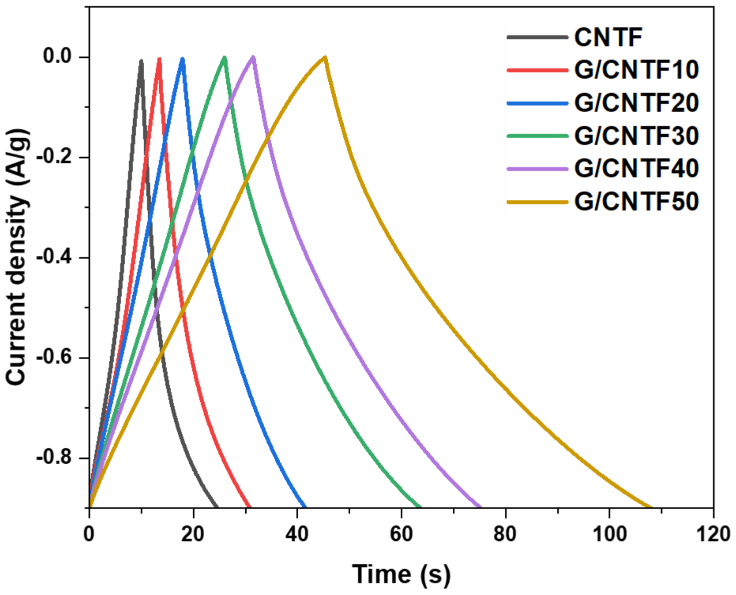

Figure S16. GCD profiles of CNTF and G-CNTF series at the current density of 1 A/g.

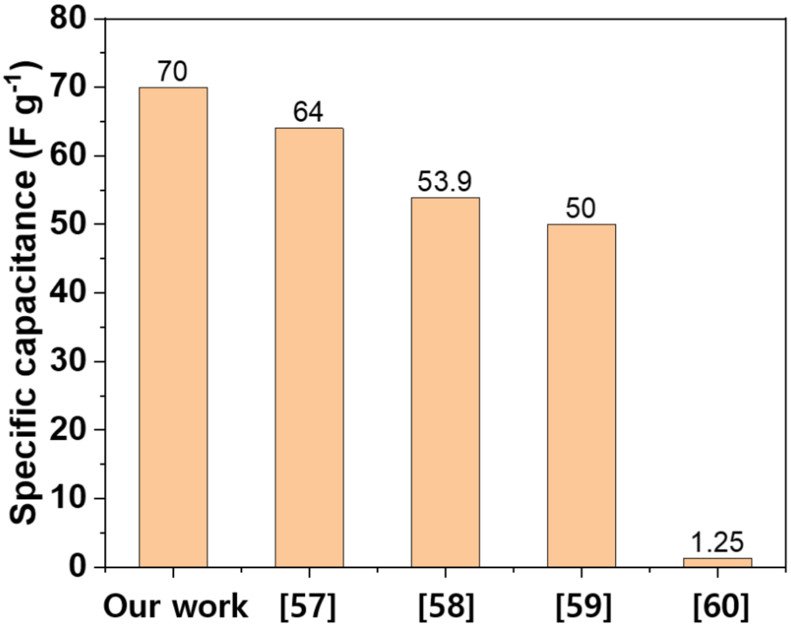

Figure S17. The specific capacitance of G-CNTF with a 50% GO contents compared to the other reported GO/CNT composite fibers.

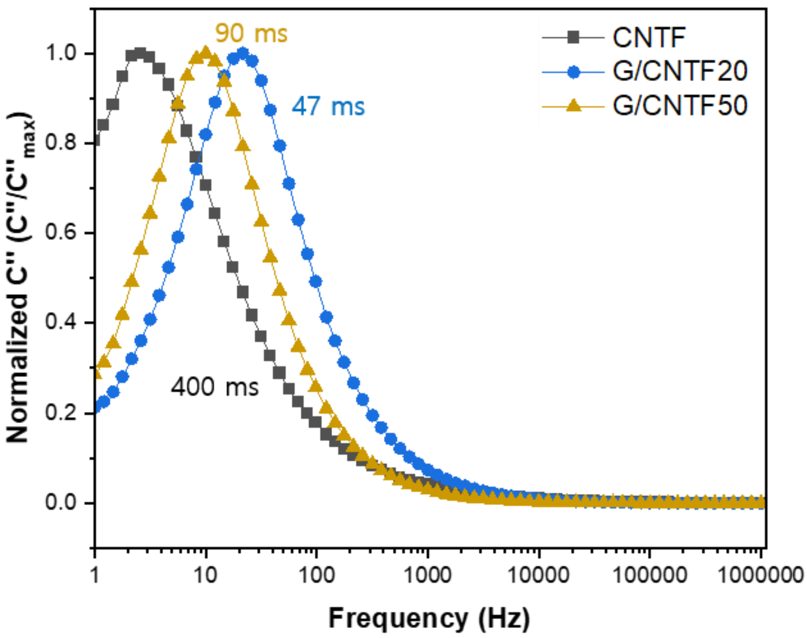

Figure S18. Normalized imaginary capacitances of CNTF, G/CNTF20, and G/CNTF50 to calculate the relaxation time.

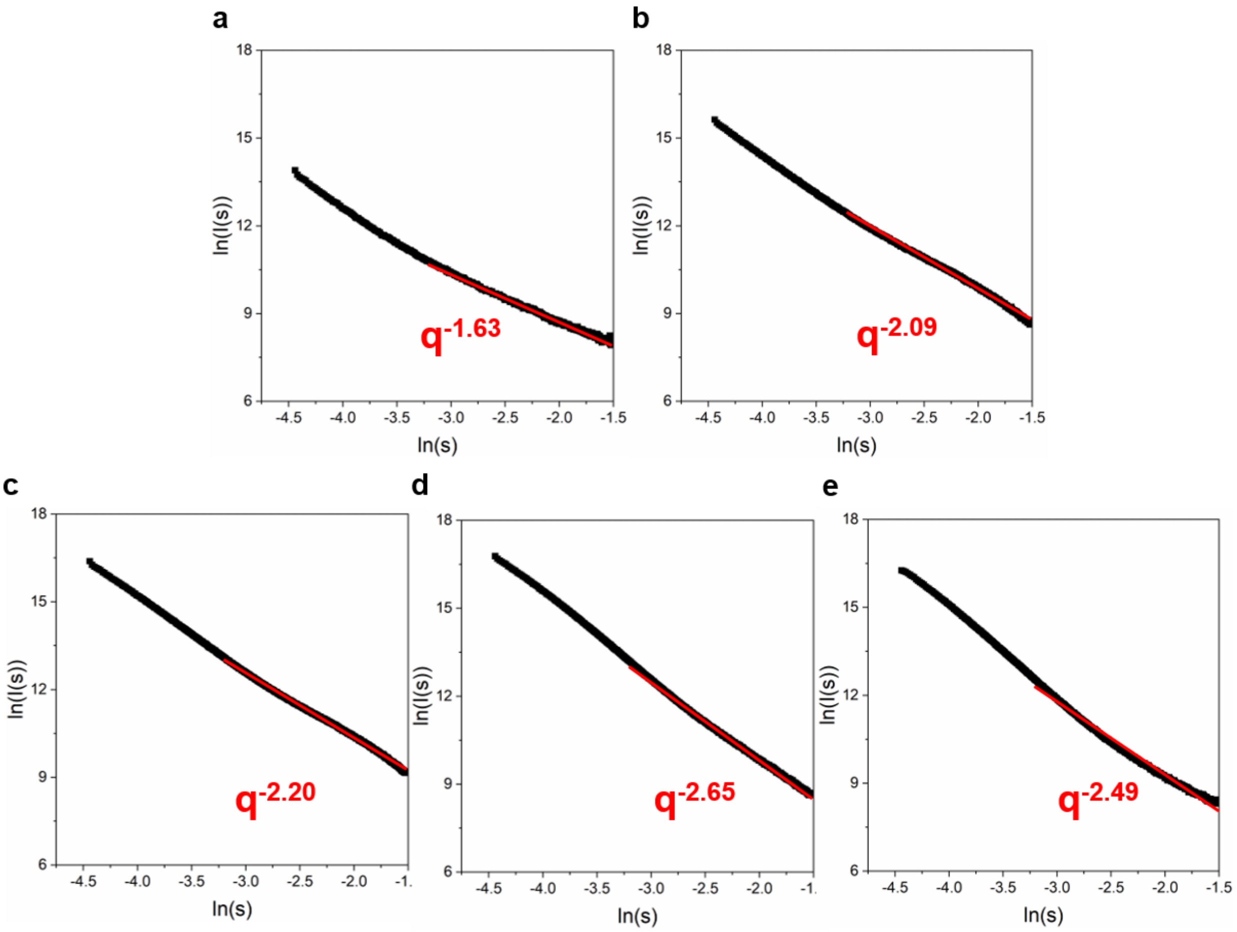

Figure S19. ln(I(s)) ~ ln(s) plots and their slope of (a) pristine CNT fiber and G/CNT fiber with (b) 10 %, (c) 20 %, (d) 30 % and (e) 40 % of GO contents.

Table S1. Specific strength, modulus, and electrical conductivity of CNT and G-CNT fibers.

GO contents (%)	0	5	10	20	30	40	50
Specific Strength (N tex^{-1})	2.05	2.66	3.01	2.41	2.11	1.89	1.56
Specific Modulus (N tex^{-1})	154	190	210	193	158	142	107
Specific Electrical Conductivity ($S\ m^2\ kg^{-1}$)	5,389	4,753	4,194	3,744	2,833	2,500	2,184

References

[1] D. E. Tsentalovich, R. J. Headrick, F. Mirri, J. Hao, N. Behabtu, C. C. Young, M. Pasquali, *ACS Appl. Mater. Interfaces* **2017**, *9*, 36189.

[2] D. E. Tsentalovich, A. W. K. Ma, J. A. Lee, N. Behabtu, E. A. Bengio, A. Choi, J. Hao, Y. Luo, R. J. Headrick, M. J. Green, Y. Talmon, M. Pasquali, *Macromolecules* **2016**, *49*, 681-689.

[3] J. Dinic, Y. Zhang, L. N. Jimenez, V. Sharma, *ACS Macro Lett.* **2015**, *4*, 804.

[4] E. S. G. Shaqfeh, G. H. Fredricksen, *Phys. Fluids A* **1990**, *2*, 7.

[5] A. W. K. Ma, F. Chinesta, T. Tuladhar, M. R. Mackley, *Rheol. Acta* **2008**, *47*, 447.

[6] Ch. Laurent, E. Flahaut, A. Peigney, *Carbon* **2010**, *48*, 2994.

[7] S. Y. Jang, S. Ko, Y. P. Jeon, J. Choi, N. Kang, H. C. Kim, H.-I. Joh, S. Lee, J. Ind. Eng. Chem. **2017**, *45*, 316.
